# Percutaneous Microwave Ablation of Hepatocellular Carcinoma with “Double Fusion” Technique: Technical Note and Single-Center Preliminary Experience

**DOI:** 10.3390/diagnostics13142349

**Published:** 2023-07-12

**Authors:** Pierpaolo Biondetti, Velio Ascenti, Anas Shehab, Anna Maria Ierardi, Serena Carriero, Carolina Lanza, Salvatore Alessio Angileri, Giuseppe Guzzardi, Gianpaolo Carrafiello

**Affiliations:** 1 Department of Diagnostic and Interventional Radiology, Foundation IRCCS Cà Granda-Ospedale Maggiore Policlinico, Via Francesco Sforza 35, 20122 Milan, Italy; 2 Postgraduation School in Radiodiagnostics, Università Degli Studi di Milano, Via Festa del Perdono 7, 20122 Milan, Italy; 3 Fellowship in Interventional Radiology, Foundation IRCCS Cà Granda-Ospedale Maggiore Policlinico, Via Francesco Sforza 35, 20122 Milan, Italy; 4 Unit of Interventional Radiology, Department of Radiology, Ospedale Maggiore della Carità, Corso Giuseppe Mazzini 18, 28100 Novara, Italy; 5 Università Degli Studi di Milano, Via Festa del Perdono 7, 20122 Milan, Italy

**Keywords:** fusion imaging, percutaneous thermal ablation, microwave ablation, interventional oncology, CBCT

## Abstract

Percutaneous image-guided thermal ablation is included in most society guidelines for treatment of hepatocellular carcinoma (HCC). The results of this treatment in terms of efficacy depend on the ability to precisely place the device into the target tumor. Ultrasound (US) is a commonly used imaging guidance modality for its real-time feedback. However, an accurate device deployment remains challenging in some clinical scenarios, including cases of tumors that are undetectable or not clearly visible by US. To overcome this problem, fusion imaging techniques have been developed, which combine images from different modalities. The most widely known technique combines pre-procedural contrast-enhanced computed tomography (CT) or magnetic resonance imaging (MRI) with real-time US scans. Cone beam CT (CBCT) is a technology that can provide intra-procedural cross-sectional images, which can be registered to images from other modalities, including preprocedural CT/MR scans. The aim of our study is to report the preliminary experience on percutaneous microwave ablation (MWA) of patients with HCC that were treated using the “double fusion“ technique, which combines the use of US fusion imaging and CBCT fusion imaging. We describe the technical details, feasibility, safety and short-term efficacy of this technique in a small series of eight patients with 11 HCCs.

## 1. Introduction

In the field of interventional radiology, recent technological advances have provided numerous therapy choices for people suffering from hepatocellular carcinoma (HCC), which is one of the leading causes of cancer-related fatalities worldwide [1].

Percutaneous image-guided thermal ablation has demonstrated its efficacy in the literature in the treatment of selected patients with HCC, and is today included in major society guidelines [2].

Microwave ablation (MWA) relies on generation of high-frequency electromagnetic waves, agitation and friction of water molecules, ultimately producing heat and coagulation necrosis [3]. MWA has demonstrated some advantages over other ablation modalities and in particular radiofrequency ablation including higher penetration in dehydrated or charred tissues, the ability to reach extremely high temperatures and larger ablation volumes in a shorter time, and less susceptibility to the “heat sink effect” with a mechanism that does not require the use of ground pads [4,5,6].

Safety and efficacy of percutaneous MWA depend on a precise placement of the antenna within the tumor, which guarantees that the ablation volume totally encompasses the target tumor volume, at the same time sparing delicate anatomical structures and non-tumoral parenchyma [7,8]; imaging guidance has a crucial role for the achievement of this task. Among imaging modalities, ultrasound (US) is the most commonly used for liver ablation and widely appreciated for its real-time anatomical assessment.

Nevertheless, despite the advances in imaging technology, the correct placement of the device by US remains a challenge in clinical practice in some cases, including tumors with poor sonographic conspicuity, deep tumors and large patients.

These problems have spurred the development of fusion imaging methods that capitalize on the strengths of different imaging modalities by overlapping different imaging datasets, including datasets obtained by different imaging modalities and/or with different timing, into a single composite imaging dataset [9]. One of the most widely known and used techniques is the registration of pre-procedural computed tomography (CT) or magnetic resonance imaging (MR) to real-time US scans, which was demonstrated to be capable of advancing efficiency and precision of percutaneous thermal ablation in particular by achieving a better lesion targeting [10]. Nevertheless, co-registration of images does not always allow a perfect fusion between the datasets and mistargeting continues to occur [11].

Cone beam CT (CBCT) is a technology that is capable of obtaining volumetric datasets through the rotation of a C-arm, with an X-ray tube and a flat panel detector mounted at its ends, around the patient that is lying on the angiographic table; data can then be visualized as cross-sectional images, today similar in quality to conventional CT [12]. CBCT images can also be registered to pre-procedural CT/MR, and the utility of CBCT fusion imaging for ablation has been reported [13,14].

In this study, we hypothesized that the use of two different fusion imaging techniques during a single session of image-guided percutaneous thermal ablation of HCC could improve treatment safety and efficacy. Therefore, we performed some liver ablation procedures using a “double fusion” technique, consisting in the application of US fusion imaging followed by CBCT fusion imaging, as described below.

The objective of this study is to report our preliminary experience in terms of technical feasibility, safety and efficacy of treatment of patients with HCC undergoing percutaneous image-guided MWA with a “double fusion” technique.

## 2. Materials and Methods

### 2.1. Study Population

In the present study, performed in accordance with the ethical standards of the institutional research committee and with the 1964 Helsinki declaration, we retrospectively reviewed patients with HCC that were treated percutaneous MWA using a “double fusion” technique between September 2021 and February 2023.

All patients included in the study met the following inclusion criteria: age ≥ 18 years; solitary HCC measuring ≤3.5 cm or ≤3 HCC lesions each measuring ≤3.0 cm; no radiologic evidence of vascular invasion or extra-hepatic disease; target tumor not visible at US; Child–Pugh grade A or B; availability of pre-procedural imaging (CT/MR) performed within 2 months from procedure date; availability of ≥1-month radiological follow-up.

Indication to treatment was given by a multidisciplinary board including interventional radiologists, hepatologists and surgeons. Eligibility for ablation was determined on the basis of standard criteria such as disease stage, comorbidity and patient age.

Indication, risks and benefits of the procedure were discussed with all patients prior to treatment, and informed consent was obtained.

The following variables were collected for each patient after consultation of clinical records and preprocedural imaging: age; sex; tumor number, location and dimension (maximum axial diameter).

**Figure 1:**
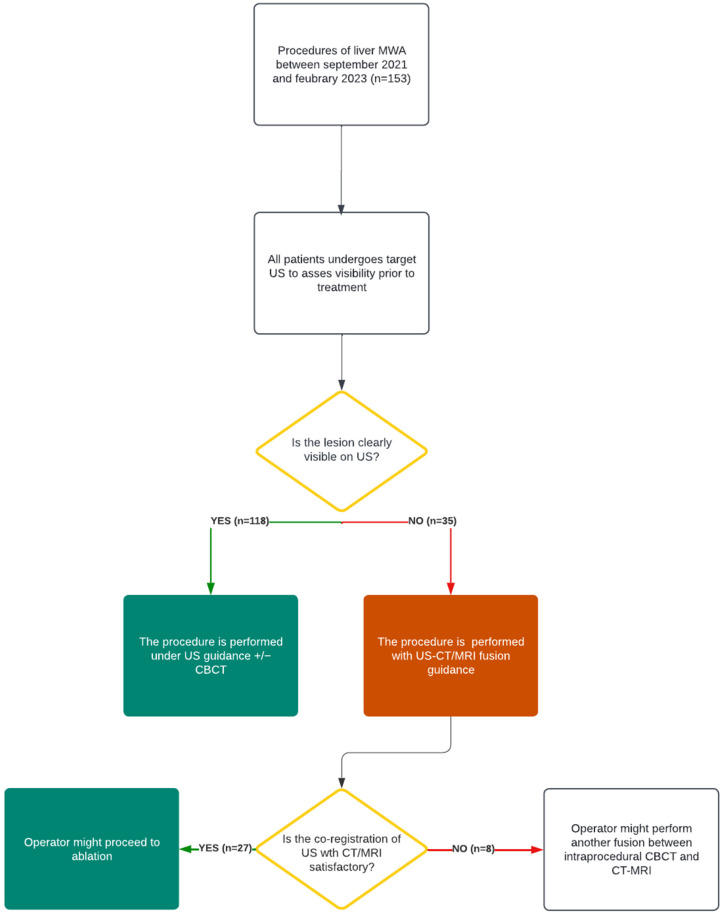
Decision-making process flowchart; MWA: microwave ablation; US: ultrasound; CBCT: cone beam computer tomography; *n*: number of procedures.

A flowchart of the decision-making process that was adopted during this study is presented in Figure 1.

### 2.2. Procedure

Risks and benefits of the proposed treatment were discussed with each patient before procedure and informed consent was obtained.

All procedures were performed in a sterile environment under moderate–deep sedation with the assistance of an anesthesiologist. In particular, sedation was mild during the antenna placement and deeper during the ablation. This distinction between different levels of sedation allows the operator to work with a cooperating patient (i.e., able to remain still and hold his or her breath) during the c-arm rotation. When the operator reaches the lesion with the antenna, proper time is given to the anesthesiologist to deepen the sedation. Heart rate, electrocardiographic trace, oxygen saturation, respiratory frequency and blood pressure were continuously monitored throughout the procedure.

Local anesthesia at the entrance site was also performed using 2% lidocaine. Antibiotic prophylaxis was achieved with intravenous administration of 2 g of cefazolin sodium (Ancef, SmithKline Beecham Pharmaceuticals, Philadelphia, PA, USA) before procedure.

Each patient was positioned supine on the angiographic table in order to have the same intraprocedural decubitus as that of preprocedural CT/MR, thus minimizing the chances of anatomical distortion when applying fusion imaging.

The ablation system device used consisted of a microwave generator (Emprint™ HP Ablation System, Medtronic, Dublin, Ireland) capable of producing up to 150 W of power at 2.45 GHz, connected by coaxial cable to a 13-gauge straight microwave antenna with a length of 15 or 20 cm. The antenna was continuously perfused with a saline solution to prevent overheating.

### 2.3. “Double Fusion” Technique

**Figure 2:**
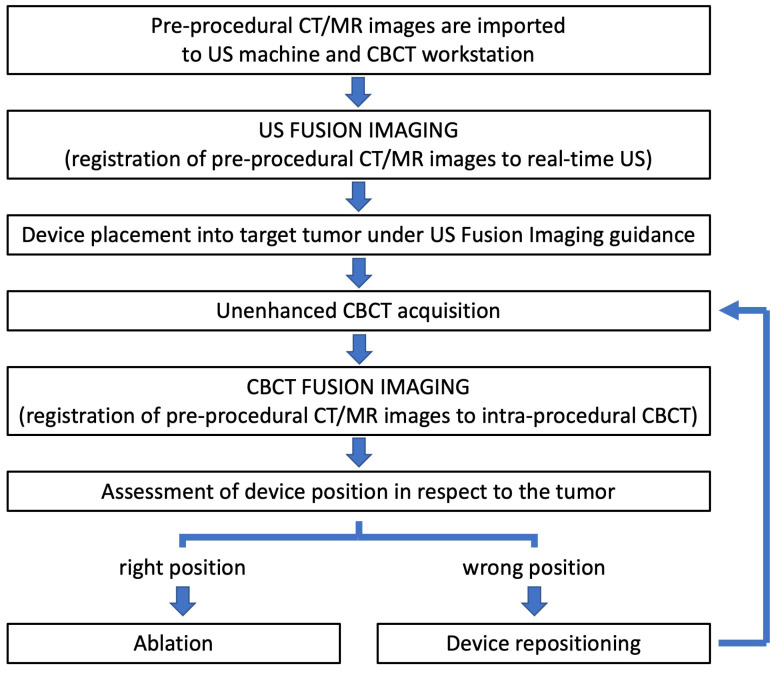
“Double fusion” technique procedural steps. CBCT: cone beam computer tomography; CT: computer tomography; MR: magnetic resonance imaging; US: ultrasound.

The following actions were performed for all patients, as shown in Figure 2.

First, preprocedural CT/MR images were transferred both to the ultrasound scanner (EPIQ, Philips Healthcare, Amsterdam, The Netherlands) and to the angiograph (Azurion Clarity, Philips Medical Systems, Best, The Netherlands) workstation, where tumor segmentation was performed (Segmentation Tool, Philips Medical System).

**Figure 3:**
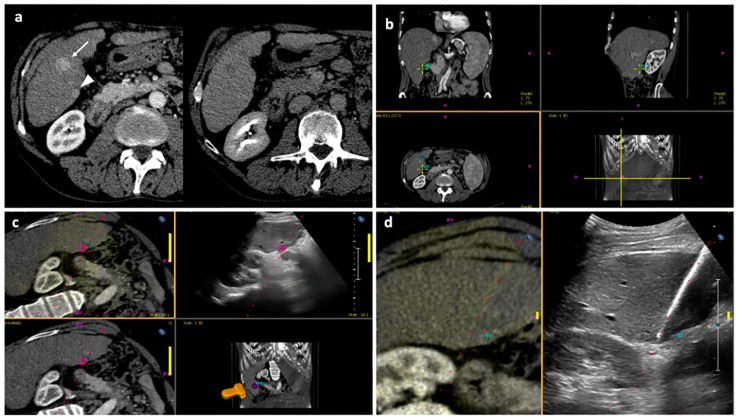
“Double fusion” technique—Part 1. (**a**) Pre-procedural axial CT images in the arterial and delayed phase showing a larger nodule with wash-in and wash-out consistent with HCC in segment V (white arrow), which was visible at US examination, and a smaller posterior subcapsular nodule with the same features (white arrowhead), which was not visible at US. The patient was scheduled for percutaneous MWA of the larger nodule under US guidance and of the smaller nodule with US fusion imaging guidance. (**b**) The smaller tumor is selected on the US scanner by visualization of pre-procedural CT images in the axial, coronal and sagittal planes. (**c**) Once registration of pre-procedural CT images to real-time US images are performed, the fused images are shown at the same time on the left upper quadrant of the US screen, simple US images are displayed on the right upper quadrant, CT images are shown on the left lower quadrant and the US probe 3D position is shown on the right lower quadrant. In this image, the smaller target tumor is visualized as a pink circle. (**d**) When US fusion imaging quality is judged adequate, the MWA antenna is placed into the target tumor; fused images are shown on the left while simple US images are shown on the right. Abbreviations—CBCT: cone-beam computed tomography; CT: computed tomography; MWA: microwave ablation; US: ultrasound.

On the US scanner, preprocedural CT/MR-imported images were visualized in the axial, coronal and sagittal planes in order to set the target. The US fusion technique was performed via electromagnetic tracking system. The US machine was connected to a magnetic field generator, to a position sensor and to a position sensor unit. The magnetic field generator created a magnetic field, inducing currents in the position sensor that was mounted on the US transducer. As the US probe moved, the magnitude of electrical current in the position sensor changed with respect to the magnetic field; with this information, the position sensor unit calculated the exact location of the position sensor and thus determined the direction and position of the US transducer. The coregistration between pre-procedural images and real-time US images was performed by automatic vessel-based registration: an ultrasound liver “volume” was obtained by swiping the US probe with the patient holding breath, and the vessels within the US volume were automatically matched to the vessels of the CT/MR by the software (PercuNav System, Philips Medical Systems). At this point, the registration quality between preprocedural CT/MR images and real-time US images was evaluated, and manual adjustments were performed if needed. Once the US fusion imaging quality was judged adequate, the best route to the tumor was planned, local anesthesia at the entrance site was performed and the microwave antenna was placed into the tumor under US fusion imaging guidance. An example of the technique used in the first part of procedure, from the procedure start to the placement of antenna under US fusion imaging guidance, is illustrated in Figure 3.

Once the antenna was positioned, an unenhanced CBCT (XperCT, Philips Image Guided Therapy) was performed before ablation, with the patient possibly holding breath, using an open arc trajectory as previously described [14]. Each CBCT acquisition was acquired through a 240-degree rotation of the C-arm around the patient in 5.2 s. The obtained X-ray projections were automatically transferred to a 3D workstation (XtraVision, Philips Image Guided Therapy) resulting in a volumetric reconstruction after automatic Feldkamp back projection was performed, all in less than 10 s.

CBCT fusion imaging was performed by manual registration of unenhanced intraprocedural CBCT images, showing the position of the antenna, to preprocedural contrast-enhanced CT/MR images, in which the tumor was visible and segmented. In this way, the spatial relationship between the antenna and the target tumor was visualized in the axial, coronal and sagittal planes. Moreover, using a dedicated software (XperGuide System, Philips Medical Systems), a virtual antenna was placed exactly over the real one, and the predicted ablation volume was displayed at its tip based on the chosen ablation power and time, according to the manufacturer data, all in order to confirm adequate tumoral coverage.

In those cases where the operator decided to change the antenna position based on the result of this process, a new unenhanced CBCT was performed with the antenna in the new position, and CBCT fusion imaging was again applied.

When the position of the antenna was judged satisfactory, the lesion was ablated under US surveillance, and track ablation was performed at the end of treatment.

Immediate complications were assessed by US and/or a final CBCT.

**Figure 4:**
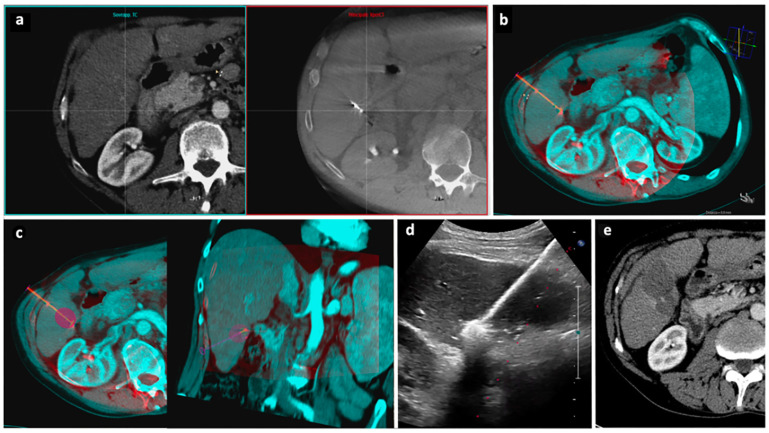
“Double fusion” technique—Part 2. After the MWA antenna is placed into the tumor (Figure 3), an unenhanced CBCT is acquired and CBCT fusion imaging is performed. (**a**) The manual registration process begins with the side-by-side visualization on the workstation of preprocedural CT images on the left and intraprocedural CBCT images on the right. (**b**) CT images are superimposed to CBCT images to assess the spatial relationship between the tumor and the MWA antenna; a virtual antenna is placed exactly over the real one. (**c**) The predicted ablation volume is displayed as a purple area at the tip of the virtual antenna based on the selected ablation power and time, according to the MWA device manufacturer data; the predicted ablation volume is here assessed on the axial (**left**) and coronal (**right**) planes. (**d**) If the antenna position is judged satisfactory by CBCT fusion imaging, ablation is carried out under US guidance. (**e**) Follow-up axial CT images acquired 1 month after procedure demonstrate complete response. Abbreviations—CBCT: cone beam computed tomography; CT: computed tomography; MWA: microwave ablation; US: ultrasound.

An example of the procedural steps performed for the second part of procedure, from the use of CBCT fusion imaging to the end, is illustrated in Figure 4.

All patients underwent at least two abdominal US in the 2–3 h after procedures to assess the presence of complications.

The following variables were collected for each procedure: antenna repositioning; ablation power; procedural time (from patient’s entrance into the angiographic suite to the last CBCT scan performed); technical feasibility, defined as the successful technical application of the “double fusion” imaging technique protocol.

### 2.4. Outcome

Each patient underwent radiological follow-up contrast-enhanced CT at 1 month.

Safety was measured by registering complications during procedure, after procedure and at follow-up; complications were recorded and classified according to the CIRSE Classification System For Complications [15].

Efficacy was measured at radiological follow-up in the form of absence of local residual disease [16].

## 3. Results

Between September 2021 and February 2023, 8 patients (all males, age mean 66 years, range 55–87 years) with 11 HCCs were treated with percutaneous image-guided MWA using “double fusion” technique.

Target tumors were located in Segments 6 (*n* = 5), 5 (*n* = 1), 7 (*n* = 1), 4 (*n* = 2), and 8 (*n* = 2).

Mean target tumor size was 14 mm (range 10–23 mm).

Mean procedural time was 76 min. For patients with two lesions, the mean time was 85 min, while for those with one lesion it was 73 min.

The antenna was repositioned based on the information obtained from CBCT fusion imaging in 5/8 procedures. Of these 5, the antenna was repositioned one, one, two, three, and two times, respectively, before ablation.

Six lesions were treated with a power of 150 W and five with a power of 100 W.

The procedure was technically carried out with success in all patients.

No complications were recorded.

At 1-month follow-up clinical success was achieved in 7/8 patients (Table 1).

## 4. Discussion

Percutaneous image-guided thermal ablation has demonstrated good results in the treatment of selected patients with HCC and is today inserted in all major societies guidelines. A key factor for treatment success is a precise placement of the ablation device into the target tumor. Despite the continuous technological advancement of devices and imaging modalities, tumor targeting remains a challenge in some clinical scenarios, including those cases where tumors have poor sonographic conspicuity at US examination. Fusion imaging techniques, which are based on coregistration of datasets obtained by different modalities and/or at different timing, allow to overcome this problem in many cases.

In this study, we report our preliminary experience on patients with HCCs that were not visible by US, treated with percutaneous image-guided MWA using a “double fusion” technique, which combines US fusion imaging and the less known CBCT fusion imaging.

The technique was successfully applied in all patients with satisfactory results in terms of safety and short-term outcome. We observed no complications and complete local tumor response at one month in 7/8 patients.

The idea of combining two different fusion techniques in a single treatment session comes from the purpose of minimizing the chance of error that every imaging fusion technique intrinsically carries, especially when datasets are derived from different imaging modalities.

US fusion imaging is today the most widely known and used for intervention as it combines clear visualization and localization of targets with poor sonographic conspicuity given by CT/MR, at the same time maintaining the advantages derived from real-time US. Nevertheless, the precision of co-registration of CT/MR images to US can be limited by anatomical distortion and respiratory movement, and even a subcentimetric misregistration can affect outcome when targets are often small like HCC.

For this reason, given our experience with CBCT as imaging guidance modality in ablation, we decided to add a further method of fusion imaging that could offer a cross-sectional anatomical evaluation of the spatial relationship between microwave antenna, shown by the intra-procedural unenhanced CBCT images, and tumor, shown by preprocedural contrast-enhanced CT/MR images, before activating the device. For optimal CBCT image analysis, it is important that the patient is in apnea during the c-arm rotation, an easily achievable goal with our sedation protocol (see Section 2.2 Procedure). Co-registration is performed manually with target organ profiles as reference; it is advisable to try to obtain image registration in the immediate vicinity of the lesion to have the best quality of fusion in that area.

This additional step in respect to simple US fusion imaging offered the possibility of a second-level check of the antenna position and of adjustments when needed, which occurred in more than half of the patients: the antenna was repositioned based on the information given by CBCT fusion imaging in 5/8 treatment sessions. This proportion is lower than that reported by Floridi et al. in a study focused on CBCT fusion imaging, where the repositioning rate was 73% [14].

**Figure 5:**
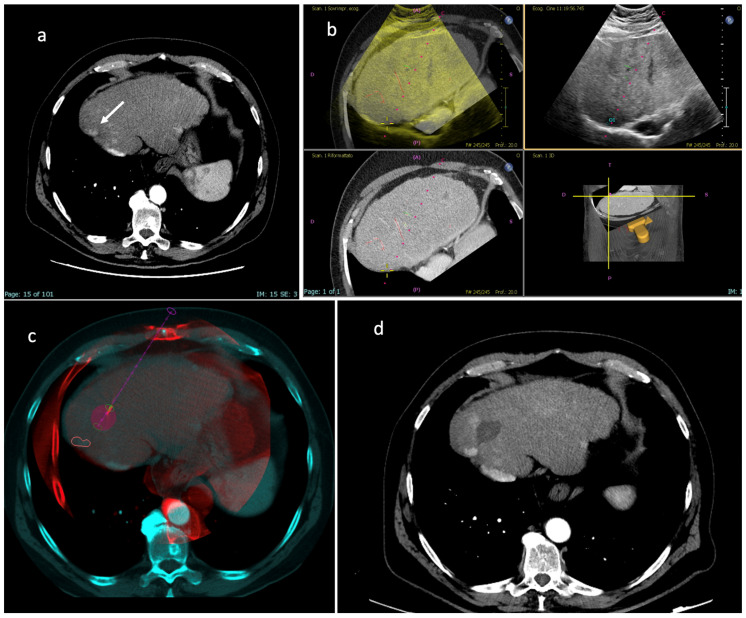
Missed target; (**a**) Axial CT image in the arterial phase showing a hypervascular nodule in S8, postero-lateral to an ablation volume from previous treatment, consistent with recurrent disease (white arrow). (**b**) US fusion imaging is performed by registrating pre-procedural CT images to real-time US images, and the antenna is placed within the target tumor. (**c**) CBCT fusion imaging indicates that the antenna and the predicted ablation volume (purple) are distant from the tumor (red contour), differently from what is suggested by US fusion imaging. Since the registration quality between US real-time images and pre-procedural CT images appeared good, the operator decided to ablate without repositioning the antenna. (**d**) CT axial image in the arterial phase at 1-month follow-up resulted comparable to intraprocedural CBCT fusion imaging, demonstrating that the nodule was missed.

Since the precision of manual coregistration performed in CBCT fusion imaging can also be affected by anatomical distortion between the datasets and movement during CBCT acquisition, it is possible, but rare, at least in our experience, to obtain very different information from the two fusion imaging guidance techniques. Giving priority to the information provided by one over the other in this situation can be challenging. In the single patient in which no tumor control was observed at 1-month follow-up, the antenna seemed perfectly positioned with US fusion imaging, while it resulted completely out of the tumor following CBCT imaging information; since the registration quality appeared superior for US fusion imaging, the operator performed the ablation without moving the antenna, but CT images at 1 month almost perfectly resemble intraprocedural CBCT fusion images, with a complete miss (Figure 5). In this case, CBCT fusion imaging resulted to be superior, but the question of the imaging modality to give priority to when modalities provide different information remains open; generally, one should prefer the modalities that achieve the best registration, but this is hard to assess in a reasonable amount of time during procedure.

What is clear is that the “double fusion” technique could achieve its maximum advantage in the difficult clinical scenario of a patients with a tumor of poor sonographic conspicuity, in which US fusion imaging quality is suboptimal. In the era of personalized medicine, and in the context of availability of multiple imaging guidance techniques, it is reasonable that in the future, the choice of the modality of guidance to be used for each case will depend on patient’s and tumor features rather than depending on the operator’s preference, like today sometimes still happens [17,18].

The drawback of the technique is the longer procedural time when compared to US fusion only or to conventional US-guided thermal ablation, even if the procedure times reported in this experience could be partially explained by the “learning curve” in these first cases. Nevertheless, a longer procedural time is justified if it can potentially improve outcome and furthermore is applied in selected cases.

A brief comparison may be made between the technique proposed in this study, at least initially technically demanding, and other available modern guidance techniques for ablation, including navigation systems. Navigational systems, most commonly used with CT, require an initial contrast-enhanced study and the absence of movement of the target region between the planning CT and the device placement; this is today commonly achieved with general anesthesia and temporary respiratory disconnection or high-frequency jet ventilation, while respiratory gating is more complex and real-time deformation models are still developing. Of note, the “double fusion” technique is applicable in patients under moderate–deep sedation, allowing to avoid exclusion from treatment of those patients that would not meet requirements for general anesthesia. Moreover, the technique described in this preliminary experience does not require intra-procedural contrast medium administration, which could be an advantage in selected patients.

Of note, this protocol was applied in patients under moderate–deep sedation. Even if technically demanding and at least initially time-consuming, maybe this approach is more effective than that of modern navigation systems.

This study, as a preliminary experience, is limited by the low number of subjects. Moreover, follow-up time is short and the outcome of patients treated with this technique is not compared to outcome of a population treated with different imaging guidance techniques.

## 5. Conclusions

In conclusion, percutaneous image-guided MWA of patients with HCC using the “double fusion” technique, which combines US fusion imaging and CBCT fusion imaging, seems to be technically feasible, safe and of satisfactory efficacy. Of course, no further conclusions can be stated from this preliminary experience with low patient number, and further studies with larger populations and follow-up are needed. Nevertheless, this technique could offer its best results in selected patients with tumors which are not visible at US and in which US fusion imaging quality seems non-reliable.

## Figures and Tables

**Table 1:** Patient characteristics. Abbreviations—FU: follow-up; C.R.: complete response; N.R.: non-response.

Patient Number	Patient Age	Tumor Dimension	Tumor Segment	Ablation Power (Watts)	Procedural Time	Antenna Repositioning	1-Month FU
1	61	11.3	6	100	75 min	Yes	C.R.
2	66	12.5	8	150	75 min	Yes	C.R.
3	66	13	4	100	65 min	No	C.R.
4	65	18; 23	4, 8	150; 150	80 min	No	C.R.
5	68	14	7	100	60 min	No	N.R.
6	87	11	6	150	75 min	Yes	C.R.
7	55	17; 8	5, 6	100; 150	90 min	Yes	C.R.
8	58	17; 10	6, 6	100; 150	90 min	Yes	C.R.

## Data Availability

Data were obtained through consultation of electronical medical record.

## References

[1] Chidambaranathan-Reghupaty S., Fisher P.B., Sarkar D. (2021). Hepatocellular carcinoma (HCC): Epidemiology, etiology and molecular classification. Adv. Cancer Res..

[2] European Association for the Study of the Liver (2018). European Association for the Study of the Liver. EASL Clinical Practice Guidelines: Management of hepatocellular carcinoma. J. Hepatol..

[3] Carrafiello G., Laganà D., Mangini M., Fontana F., Dionigi G., Boni L., Rovera F., Cuffari S., Fugazzola C. (2008). Microwave tumors ablation: Principles, clinical applications and review of preliminary experiences. Int. J. Surg..

[4] Facciorusso A., Di Maso M., Muscatiello N. (2016). Microwave ablation versus radiofrequency ablation for the treatment of hepatocellular carcinoma: A systematic review and meta-analysis. Int. J. Hyperthermia.

[5] Lu D.S., Raman S.S., Limanond P., Aziz D., Economou J., Busuttil R., Sayre J. (2003). Influence of large peritumoral vessels on outcome of radiofrequency ablation of liver tumors. J. Vasc. Interv. Radiol..

[6] Yang D., Converse M.C., Mahvi D.M., Webster J.G. (2007). Measurement and analysis of tissue temperature during microwave liver ablation. IEEE Trans. Biomed. Eng..

[7] Kim Y.S., Rhim H., Cho O.K., Koh B.H., Kim Y. (2006). Intrahepatic recurrence after percutaneous radiofrequency ablation of hepatocellular carcinoma: Analysis of the pattern and risk factors. Eur. J. Radiol..

[8] Wang X., Sofocleous C.T., Erinjeri J.P., Petre E.N., Gonen M., Do K.G., Brown K.T., Covey A.M., Brody L.A., Alago W. (2013). Margin size is an independent predictor of local tumor progression after ablation of colon cancer liver metastases. Cardiovasc. Intervent. Radiol..

[9] Appelbaum L., Mahgerefteh S.Y., Sosna J., Goldberg S.N. (2013). Image-guided fusion and navigation: Applications in tumor ablation. Tech. Vasc. Interv. Radiol..

[10] Song K.D., Lee M.W., Rhim H., Cha D.I., Chong Y., Lim H.K. (2013). Fusion imaging-guided radiofrequency ablation for hepatocellular carcinomas not visible on conventional ultrasound. AJR Am. J. Roentgenol..

[11] Lim S., Lee M.W., Rhim H., Cha D.I., Kang T.W., Min J.H., Song K.D., Choi S.Y., Lim H.K. (2014). Mistargeting after fusion imaging-guided percutaneous radiofrequency ablation of hepatocellular carcinomas. J. Vasc. Interv. Radiol..

[12] Orth R.C., Wallace M.J., Kuo M.D., Technology Assessment Committee of the Society of Interventional Radiology (2009). C-arm cone-beam CT: General principles and technical considerations for use in interventional radiology. J. Vasc. Interv. Radiol..

[13] Abdel-Rehim M., Ronot M., Sibert A., Vilgrain V. (2015). Assessment of liver ablation using cone beam computed tomography. World J. Gastroenterol..

[14] Floridi C., Radaelli A., Pesapane F., Fumarola E.M., Lecchi M., Agostini A., Giovagnoni A., Carrafiello G., Wood B. (2017). Clinical impact of cone beam computed tomography on iterative treatment planning during ultrasound-guided percutaneous ablation of liver malignancies. Med. Oncol..

[15] Filippiadis D.K., Binkert C., Pellerin O., Hoffmann R.T., Krajina A., Pereira P.L. (2017). Cirse Quality Assurance Document and Standards for Classification of Complications: The Cirse Classification System. Cardiovasc. Intervent. Radiol..

[16] Puijk R.S., Ahmed M., Adam A., Arai Y., Arellano R., de Baère T., Bale R., Bellera C., Binkert C.A., Brace C.L. (2021). Consensus Guidelines for the Definition of Time-to-Event End Points in Image-guided Tumor Ablation: Results of the SIO and DATECAN Initiative. Radiology.

[17] D’Onofrio M., Beleù A., Gaitini D., Corréas J.M., Brady A., Clevert D. (2019). Abdominal applications of ultrasound fusion imaging technique: Liver, kidney, and pancreas. Insights Imaging.

[18] Carriero S., Della Pepa G., Monfardini L., Vitale R., Rossi D., Masperi A., Mauri G. (2021). Role of Fusion Imaging in Image-Guided Thermal Ablations. Diagnostics.

